# Biochemical Profiling of Histone Binding Selectivity of the Yeast Bromodomain Family

**DOI:** 10.1371/journal.pone.0008903

**Published:** 2010-01-26

**Authors:** Qiang Zhang, Suvobrata Chakravarty, Dario Ghersi, Lei Zeng, Alexander N. Plotnikov, Roberto Sanchez, Ming-Ming Zhou

**Affiliations:** Department of Structural and Chemical Biology, Mount Sinai School of Medicine, New York, New York, United States of America; Texas A&M University, United States of America

## Abstract

**Background:**

It has been shown that molecular interactions between site-specific chemical modifications such as acetylation and methylation on DNA-packing histones and conserved structural modules present in transcriptional proteins are closely associated with chromatin structural changes and gene activation. Unlike methyl-lysine that can interact with different protein modules including chromodomains, Tudor and MBT domains, as well as PHD fingers, acetyl-lysine (Kac) is known thus far to be recognized only by bromodomains. While histone lysine acetylation plays a crucial role in regulation of chromatin-mediated gene transcription, a high degree of sequence variation of the acetyl-lysine binding site in the bromodomains has limited our understanding of histone binding selectivity of the bromodomain family. Here, we report a systematic family-wide analysis of 14 yeast bromodomains binding to 32 lysine-acetylated peptides derived from known major acetylation sites in four core histones that are conserved in eukaryotes.

**Methodology:**

The histone binding selectivity of purified recombinant yeast bromodomains was assessed by using the native core histones in an overlay assay, as well as N-terminally biotinylated lysine-acetylated histone peptides spotted on streptavidin-coated nitrocellulose membrane in a dot blot assay. NMR binding analysis further validated the interactions between histones and selected bromodomain. Structural models of all yeast bromodomains were built using comparative modeling to provide insights into the molecular basis of their histone binding selectivity.

**Conclusions:**

Our study reveals that while not all members of the bromodomain family are privileged to interact with acetylated-lysine, identifiable sequence features from those that bind histone emerge. These include an asparagine residue at the C-terminus of the third helix in the 4-helix bundle, negatively charged residues around the ZA loop, and preponderance of aromatic amino acid residues in the binding pocket. Further, while bromodomains exhibit selectivity for different sites in histones, individual interactions are of modest affinity. Finally, electrostatic interactions appear to be a primary determining factor that guides productive association between a bromodomain and a lysine-acetylated histone.

## Introduction

Chromatin packages all genomic DNA in eukaryotic cells and functions as a master regulator that governs gene transcriptional activation and silencing. Within the highly ordered structure of chromatin, the nucleosome is the basic unit that consists of DNA of 147 base pairs wrapping in two superhelical turns around a histone octamer formed by dimer of each of H3-H4 and H2A-H2B dimers. Nucleosome core particles are linked by short stretches of DNA bound to the linker histones H1 and H5 to form a nucleosomal filament that is folded into higher-order structure of chromatin fiber. Site-specific histone modifications of acetylation, methylation, phosphorylation, ubiquitination and sumoylation largely in the N- and C-terminal residues have been shown to set a dynamic stage for all DNA-based processes within the nucleus [1]. The extremely dense and versatile nature of histone modifications argues that histone signaling is far more complex in information content than cell-surface receptor signaling [2], [3]. However, our overall mechanistic understanding of histone signaling in gene regulation lags far behind that of cellular signaling.

Recent studies show that site-specific modifications of histones serve as binding sites for effector proteins and that such histone-mediated molecular interactions individually and combinatorially are linked to distinct functions in gene regulation [1], [4]. This view is supported by the discoveries of acetyl-lysine (Kac) recognition by bromodomains (BRDs) [5], [6] and methyl-lysine (Kme) binding by the “royal” family domains of chromodomains, Tudor, MBT domains [7], [8] in histone tails, as well as PHD fingers [9].

**Figure 1:**
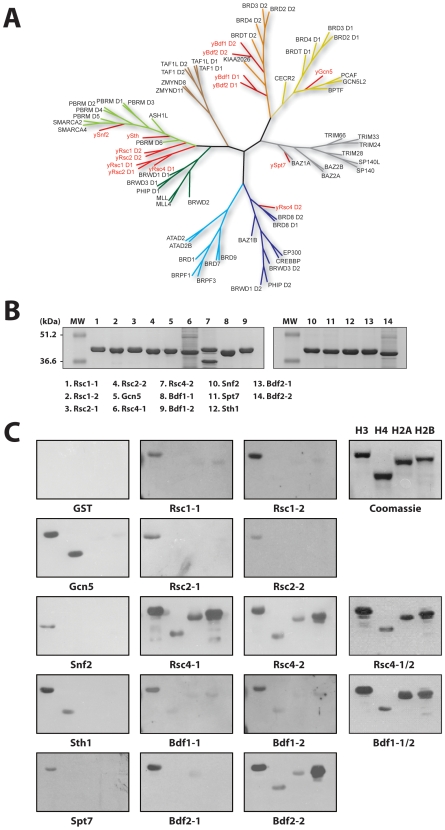
Binding of yeast BRDs to native core histones. (**A**) Sequence similarity based dendrogram of yeast (red) and human BRDs generated using the neighbor-joining method with MEGA3 [38]. Sequences of BRDs were obtained from the SMART database [39], and aligned with SMART BRDs' hidden Markov models using Hmmalign [40]. (**B**) Purity of recombinant yeast BRDs used in the binding study, shown in SDS-PAGE. (**C**) Histone overlay assay showing relative binding of yeast BRDs to four native histones from calf thymus (Roche). The GST-BRD bound to the individual native histones was visualized by Western blot using anti-GST antibody.

As a highly dynamic and reversible modification, lysine acetylation plays a key role in directing chromatin structural changes associated with gene transcription. The functional role of lysine acetylation in histone-directed chromatin biology is highlighted by a large number of bromodomain (BRD)-containing proteins and histone acetyl-transferases (HATs) (56 BRDs in 42 proteins in humans) (Figure 1A) [6]. This basic mechanism of protein-protein interactions mediated by BRD binding of acetyl-lysine supports the notion that nuclear HATs in transcription complexes are tethered to specific chromosomal sites by site-specific BRDs mediated Kac anchoring [10]–[12]. Examples of these include the assembly of multi-component chromatin remodeling complexes such as SAGA [13] and SWI/SNF [14], [15]. This mechanism may also help understand phenotypes linked to BRD deletion. For instance, the BRD module of yeast Gcn5 is required for the stable association of the SWI/SNF complex on the gPHO5 promoter [16], and is not indispensable for activation of yeast PHD5 [17]. Deletion of a BRD in HBRM in the human SWI/SNF complex causes decreased stability and loss of nuclear localization [18], [19]. BRDs of *S. cerevisiae* Bdf1 are required for sporulation and normal mitotic growth [20]. BRD deletion in members of RSC remodeling complex, causes a conditional lethal phenotype [21] or a phenotypic inhibition on cell growth [22]. Moreover, transgenic mice with lymphoid-restricted overexpression of the double BRD protein 2 develop splenic B-cell lymphoma and leukemia [23].

Despite its functional importance, our knowledge of the molecular determinants for BRD recognition of acetyl-lysine remains limited. While numerous three-dimensional structures of the bromodomain family have been experimentally determined, only a few are of the complexes of BRD bound to a lysine-acetylated-peptide of a biological binding partner [6], [24]–[28]. Nevertheless, these structural studies show that all BRDs likely adopt a conserved structural fold of a left-handed four-helix bundle (αZ, αA, αB and αC), and the ZA and BC loops at one end of the bundle form a hydrophobic pocket for Kac binding that was defined in the first BRD structure from PCAF [5]. While the BRD residues important for Kac binding are largely conserved, sequence variations in the ZA and BC loops enable discrimination of different binding targets [5], [6]. The high degree of sequence variations of amino acid deletion or insertion of the ZA and BC loops indicates that different sets of residues in these regions of a BRD dictate its ligand binding specificity by interacting with residues flanking the Kac in a target protein [6].

Molecular functions of these histone interaction domains are likely conserved from yeast to human. On the basis of sequence similarity the 14 *S. cerevisiae* BRDs seem to represent different subsets of the much larger human BRD family (Figure 1A). Because the site-specific lysine acetylation in histones, and the N- or C-terminal sequences of histones are conserved between yeast and human, we reasoned that knowledge of histone binding selectivity of the yeast BRDs can help understand the ligand binding selectivity of the human BRDs. Therefore, this unique evolutionary relationship in protein structure-function between human and yeast BRDs offers an attractive model system for us to conduct a family-wide molecular profiling of histone binding selectivity by yeast bromodomains, which we report in this study.

## Results and Discussion

### Yeast BRDs Binding to Native Core Histones

To explore histone binding activity of the BRDs encoded in *S. cerevisiae* that represent the larger family of BRDs (Figure 1A), we cloned and purified 14 yeast BRDs (yBRDs) as GST-fusion proteins (Figure 1B) and assessed their binding in an overlay assay to native core histones isolated from calf thymus (Roche) that contain post-translational modifications. In this assay, histones run in SDS-PAGE gels were transferred to nitrocellulose paper, which was incubated with GST-yBRDs or GST alone (Figure 1C). yBRDs bound to the histones on the nitrocellulose paper were visualized in Western blotting using anti-GST antibody. This binding study reveals that the yBRDs have preferential binding to different histones; some such as yGcn5 and ySnf2 bind selectively to certain histones, whereas others, i.e. yRsc2-2 and ySpt7, bind histones only weakly, if at all. While this study yields intriguing insights into selective histone binding by yBRDs, detailed interpretation can be complex. This is because multiple modifications are present simultaneously in the native histones, and yBRD binding to a particular acetylation site can be influenced positively or negatively by other modifications on the neighboring residues, thereby complicating the analysis of the histone binding selectivity.

### Site-Specific Histone Recognition by Yeast BRDs

**Figure 2:**
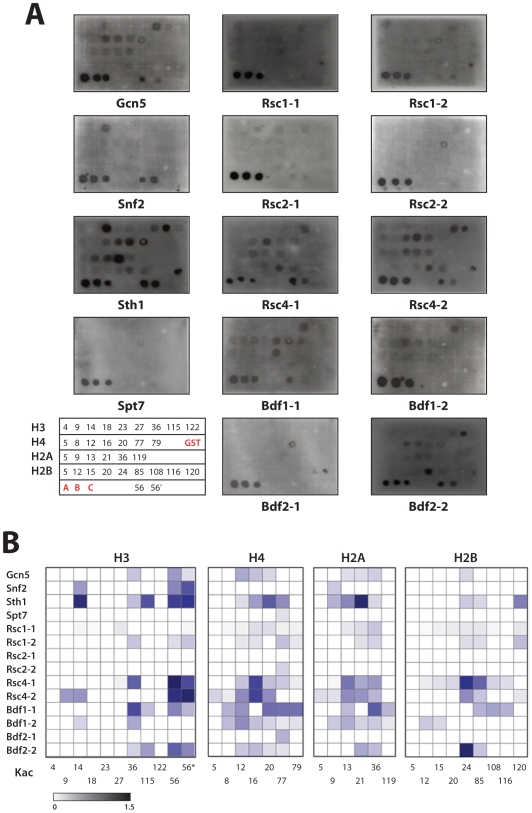
Binding of yeast BRDs to lysine-acetylated histone peptides. (**A**) Dot blot assay showing relative binding of the N-terminal biotinylated lysine-acetylated histone peptides to yeast GST-BRDs. Lysine-acetylated histone peptides were dotted on the SAM biotin capture membrane that was incubated with a GST-BRD. The bound GST-BRD was probed with anti GST-HRP conjugate. The labeling of the histone peptides is indicated in the matrix at the low left corner of the panel. The first three spots in the last row were of GST (20, 10 and 5 ng each) spotted on the membrane. 56 and 56′ refer to yeast histone H3K56ac and H3K56 peptides. (**B**) Matrix of binding preferences computed from normalized densities. The normalized values range from 0.2 (least preferred binder) to 1.5 (most preferred binder).

To circumvent this problem, we systematically evaluated yBRDs binding to 32 lysine-acetylated peptides derived from known acetylation sites in four human core histones, which are K4, K9, K14, K18, K23, K27, K36, K56, K115 and K122 of histone H3; K5, K8, K12, K16, K20, K77 and K79 of H4; K5, K9, K13, K21, K36 and K119 of H2A; and K5, K12, K15, K20, K24, K85, K108, K118 and K120 of H2B. All histone peptides consist of 15 residues with the acetyl-lysine at the center. For the N-terminal lysine acetylation sites such as H3K4ac, H4K5ac, H2AK5ac and H2BK5ac, a short segment of GGSG or GGS were added at the peptide N-terminus. All peptides were biotinylated at the N-terminus making it possible to immobilize them on a biotin capture membrane (Promega). In a dot blot assay, individual yBRDs binding to a complete set of the histone peptides spotted on the membrane were evaluated with Western blotting using anti-GST antibody (Figure 2A). To compare histone binding selectivity, we normalized signal intensity of yBRD binding of histones to that of GST spotted on the same membrane (Figure 2B).

While the results agree with those from the overlay study (Figure 1C), the dot blot assay yields more details on site-specific histone binding by the individual yBRDs. For instance, yGcn5 BRD interactions with H3 and H4 (Figure 1C) is shown predominately due to its preferred binding to H3K56ac, H4K12ac and H4K16ac over other weak binding sites (Figure 2B). Similarly, yBdf2-2 BRD binding of H2B was determined to be at H2BK24ac, and yBdf1-1 and yBdf1-2's H3 binding both at H3K36ac (Figure 2B). Notably, ySPT7 BRD binding to native H3, albeit weak, was not seen at all with the peptides. Conversely, some strong histone peptide interactions, i.e. ySTH1 BRD to H2AK21ac, yBDF1-1 BRD to H4K20ac and H2AK36ac were contrasted by their negative interactions with native H4 and H2A (Figure 1C). These seeming discrepancies in histone binding observed in the two assays may be due to one or more of the following factors: (1) lack of some lysine acetylation in native histones; (2) influences on BRD binding of histones by neighboring modifications in histones; this is exemplified by a recent report that the first BRD of the BET family protein BRDT prefers binding to histone H4 that is dually acetylated at lysines 5 and 8 [29]; and (3) some histone binding may require more than 15-mer peptides. We further observed that a few yBRDs bind specifically and equally well to certain histone peptides regardless of acetylation, e.g. ySnf2, ySth1 and yBdf2-2 to H3K56 and H3K56ac peptides. Finally, by using NMR, we confirmed a few select BRDs' histone peptide binding observed in the dot blot assay such as yGcn5 specific binding to H4K16ac, ySnf2 and ySth1 to H3K14ac, as well as ySpt7's non-binding to H3 or H3K14ac (see Figure S1). Overall, our results generally agree with previous studies of individual yeast BRDs' binding to histones using different approaches [30]-[32]. However, given that our study of yBRDs' histone binding was done under the same conditions, our results lay an important foundation to elucidate the molecular basis of hisone binding selectivity of the yeast BRDs.

### Modeled Structures of Yeast BRDs

**Figure 3:**
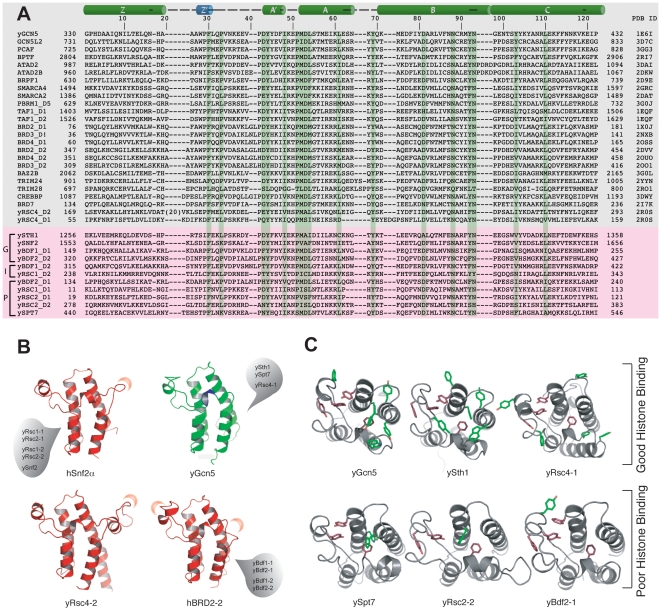
Molecular basis of histone recognition by the yeast BRDs. (**A**) Experimentally determined structures of 26 BRDs were superimposed using MODELLER [36] resulting in a structure-based multiple alignment (top). The experimental structures were used as templates for modeling 11 yeast BRDs. The yeast BRDs (bottom) are aligned to the structural and sequence similarity of both human and yeast BRD sequences. The 4-letter code with chain-ID and residue numbering of PDB templates are indicated on the left, while protein names are on the right (gray-box). The names of yeast BRDs are on the left (bottom). PDB sequences with ≥35% sequence identity are bracketed on the left. Yeast BRD sequences are grouped as good, intermediate and poor lysine-acetylated histone binders as indicated on the left (bottom). (**B**) Yeast BRD structures that deviate, due to insertion or deletion (crescents), from the archetypical yGCN5 are shown in 4 groups based on the sequence similarity. (**C**) Distribution of the aromatic residues (conserved red, non-conserved green) at the acetyl-lysine-binding pocket highlighting the differences between good and poor histone binding BRDs, as indicated.

Among the 14 BRDs in yeast, three experimental structures are available, i.e. yGcn5 BRD and yRsc4 tandem BRDs. Structures of the remaining 11 yBRDs were modeled based on available BRD structures. Structural comparison of all available structures of BRDs shows that there are primarily four distinct regions where structural variations occur in this family. The sequence conservation in the first helix Z is poor compared to that of the rest of the domain (Figure 3A) making it hard to align this region among different BRDs. This is reflected in our initial hidden Markov model (HMM) analysis that fails to define the domain boundary for the helix Z (residues 169–285) of yRsc4-2 seen in the recently determined in the structure (PDB ID 2R0S). Since we obtained soluble proteins for all 14 yBRDs, the domain boundary for yBRD constructs, which was defined based on the SMART/PFAM sequence analysis, is likely correct. With a careful consideration of the residues in the region around the helix Z, manual alignment of the 11 yBRDs was generated with respect to the 26 known BRD structures (see Materials and Methods) with an emphasis on clustered sequence neighbors at 35% sequence identity, if possible. The structures of the 14 yBRDs, therefore, can be placed in distinct groups based on amino acid insertion or deletion in the ZA loop that comprises the acetyl-lysine binding pocket (Figure 3B).

### Molecular Basis of Histone Binding Selectivity by Yeast BRDs

**Figure 4:**
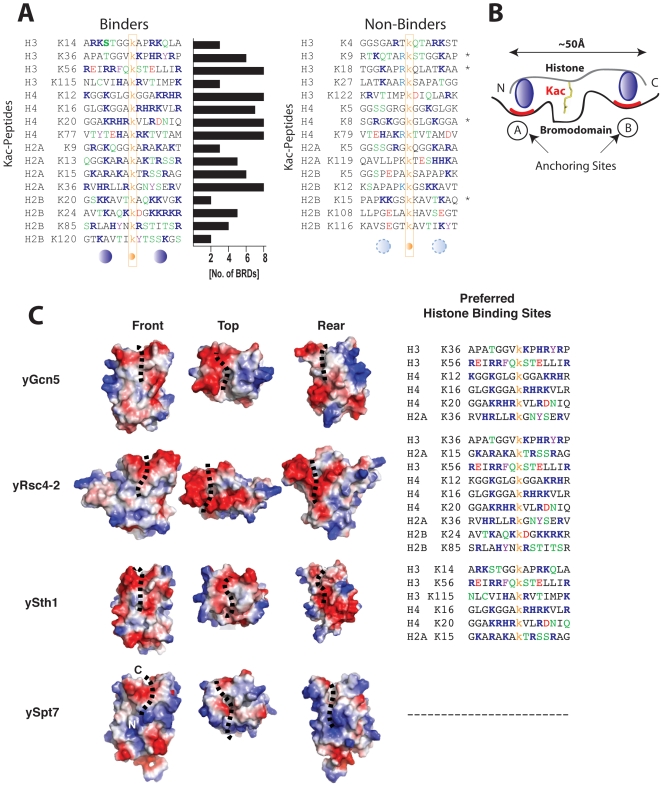
Influence of surface electrostatic potential of yeast BRDs on histone binding selectivity. (**A**) Binding of yBRDs to 15-mer acetyl-lysine (Kac)-containing histone peptides: Binders (left) show a preference for positively charged residues (blue filled circles) on either side of Kac compared to non-binders (middle, light blue dotted circle). Horizontal bars (left) represent the frequency with which each peptide binds a set of 14 yBRDs. The peptides are color-coded as the following: Kac (yellow), positively charged (blue), negatively charged (red), polar (green) and aromatic (pink) amino acids. (**B**) Cartoon illustration of the proposed mode of BRD recognition of histone sequences through two anchoring sites ‘A’ and ‘B’ flanking the Kac. (**C**) Surface electrostatic potential of yeast BRDs (left panel) corresponding to their selective binding to histone peptides (right panel). The histone peptide (black dots) is indicated on the surface of the yeast BRDs. Three views, i.e. front, top, and black are shown.

Consistent with the highly positively charged histone sequences, yeast BRDs' preferred binding sequences (binders) are rich in lysine and arginine residues on both sides of the Kac (Figure 4A, left). For example, contiguous stretches of positively charged residues in KacRHRKac in H4 (residues 16–20, where Kac is an acetylated lysine) and KacDGKKRKR in H2B (residues 24–31) are recognized by five or more BRDs, while segments with interspersed positively charged residues are more selective (Figure 4A, middle). Most non-binder sequences have few positively charged residues, and some have negatively charged residues (Figure 4A, right). Polar non-charged residues that are capable of hydrogen bonding are seen more frequently in binders than in non-binders. Conversely, proline residues adjacent to the Kac appear more frequently in non-binders than in binders indicating the importance of peptide flexibility for BRD binding. Finally, while absent in non-binders, aromatic residues such as Tyr in H3K36ac, H4K77ac or K2AK36ac, or Phe in H3K56ac are present in binder sequences for four or more BRDs. Since aromatic amino acids can engage in protein-protein interactions by aromatic stacking or cation-*π* interactions with positively charged amino acids, the yeast BRDs that interact with many histone peptides appear to have more aromatic residues at the ZA and BC loops than those that show limited histone binding (Figure 3C).

Given the high occurrence of positively charged residues flanking the Kac in the binder sequences, we reasoned that electrostatics likely plays a key role in determining histone binding selectivity by BRDs. We examined the molecular basis of BRD binding of histones with the structure models of the yBRDs (see Figure S2), which suggest that the mode of histone peptide recognition involves two anchoring sites on the BRD surface directing a peptide in either orientation (Figure 4B). As suggested by a dense negative charge patch corresponding to the central region of the ZA loop (Figure 4C), yGcn5 and yRsc4-2 likely prefer one or more positively charged residues adjacent to the Kac in the target sequence. Indeed, four of the six peptides recognized by yGCN5 BRD, six of the nine peptides by yRsc4-2 and all three peptides by yBDF1-1 contain a Lys or Arg at (Kac+/−1) positions. It is important to note that the guanidinium group of H4R17 in the H4K16ac peptide when bound to yGcn5 BRD (PDB ID 1E6I) does not, however, form any electrostatic interactions with the nearby carboxylate groups of DYYD residues in the ZA loop [27]. This suggests that electrostatics in this case is primarily needed for steering the peptide to the binding site as one also observes that peptides with a stretch of positively charged residues are preferred. This also explains the broader specificity of BRDs as each domain is able to recognize more than one peptide that shares some degree of amino acid variations.

### Structure-Based Classification of Acetyl-Lysine Binding Sites in Yeast BRDs

**Figure 5:**
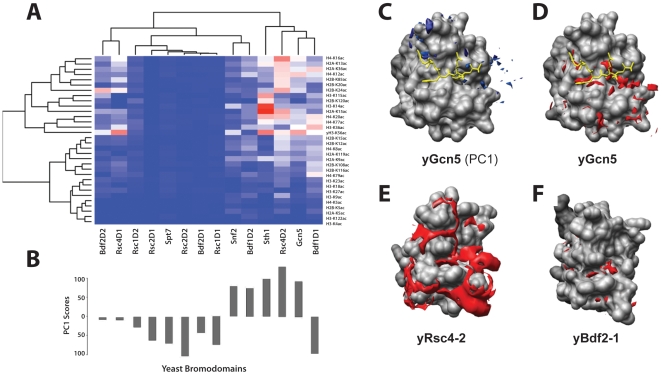
Classification of yeast BRDs based on molecular interaction properties. (**A**) Heat-map showing the clustering of yeast BRDs (left to right) and histone peptides (top to bottom) based on their relative binding affinities. The scale is relative from low affinity (blue) to high affinity (red). (**B**) Values of the first principal component (PC1) from the CPCA analysis of the Molecular Interaction Fields (MIFs) of all yeast BRDs (see text). (**C**) The blue contours refer to the regions in the BRD structure corresponding to PC1 (yGcn5 BRD is used as a representative structure). The H4K16ac peptide is shown in yellow. (**D**), (**E**), and (**F**) Molecular Interaction Field (MIF) for the N1+ (sp3 amine NH cation) probe mapped as a red contour on yGcn5, yRsc4-2, and yBdf1-2 BRDs, respectively. Note the strong N1+ signal in yGcn5 and yRsc4-2 (good histone binders) *versus* yBdf2-1 (poor histone binder).

We performed a family-wide structural analysis of all yeast BRDs to further identify structural properties that may explain their histone binding selectivity, which cannot be elucidated by sequence comparison alone. These 2 groups are clearly identifiable when the clustering is based on experimental data (Figure 5A). Molecular Interaction Field (MIF) analysis of the BRD structures was carried out to quantitatively characterize the molecular binding propensities in their peptide binding sites. Principal Component Analysis (PCA) of the MIF data showed that the first principal component (PC1) produced a clustering of BRDs very similar to that obtained using experimental data (Figure 5A–B). With the exception of yBdf1-1 BRD, all BRD with positive, or close to zero, values of PC1 show relatively high affinity for histone peptides. Conversely, BRDs with negative values of PC1 did not show binding to histone peptides. The combined MIF and PCA analysis also identified the regions in the BRD structure that contribute the most to PC1, and thus to the correct clustering (Figure 5C). As MIFs contain chemical information, it is possible to identify the probe (or a combination of probes) that account for the effect. The results for the N1+ probe indicate that binding of positively charged groups is one of the important factors in distinguishing BRDs that bind histones from those that do not bind histones (Figure 5D–F), consistent with the effect of electrostatics described above. Taken together, our study suggests that the MIF/PCA analysis of BRD structures could provide an automated means to identify BRDs with propensity for histone interaction.

### Concluding Remarks

BRDs likely all adopt the conserved left-handed four-helix bundle with the ZA and BC loops forming the acetyl-lysine binding pocket [6]. However, unlike other protein domains such as SH2 or PTB domains that bind a modified amino acid (i.e. phospho-tyrosine) in a consensus sequence in a target protein with a structurally defined ligand binding pocket [3], [33], the acetyl-lysine binding site in the BRD is composed of segments (i.e. the ZA and BC loops), which are highly flexible in three-dimensional structure, and shows high variation in geometry and sequence composition. Because of this extraordinarily high degree of variation in the ligand binding site, as well as relatively modest binding affinity (*K_d_* of tens-to-hundreds µM), it is extremely difficult to predict binding partners for the large BRD family with knowledge of only three available structures of BRD and acetyl-lysine-peptide complexes [6]. The knowledge of the structure-function relationship of any protein domain should be generated with biologically relevant ligands. The evolutionarily conserved histone binding activity of BRDs from yeast to human makes it an ideal model system for a systematic genome-wide profiling of domain/ligand selectivity by using combined experimental and computational methods.

The study we report here suggests that there are possibly three important factors that are responsible for histone binding selectivity by the BRDs. These are (1) structure and dynamics of the ZA loop of the BRD, (2) negatively charged residues at anchoring sites, and (3) presence of aromatic residues in the vicinity of binding sites are responsible for peptide recognition. The last factor is possibly the least influential one as there are some cases such as yRsc1-1, yRsc2-1 and ySnf2 BRDs where the presence of the aromatic clusters does not favor peptide binding. Moreover, adjacent modifications could also positively or negatively influence binding specificity for a given acetylation site. One example of the former is a recent report, which shows that the first BRD of the BET family protein BRDT prefers binding to histone H4 when dually acetylated at lysines 5 and 8 [29]. While negative regulation by an adjacent modification has not been reported for BRDs, it has been shown that the sequence specific histone H3 recognition by the BHC80 PHD finger binding to non-modified H3 is attenuated upon methylation of H3 lysine 4 in gene transcriptional repression [34]. Therefore, it is conceivable that a detailed understanding of the molecular basis of the histone binding selectivity would require additional structural analysis of BRDs bound to different peptides derived from their biological binding partners.

## Materials and Methods

### Cloning, Expression, and Purification of Yeast BRDs

DNA fragments encoding fourteen yeast BRDs were PCR amplified from yeast genomic DNA and cloned into the pGEX-4T-1 vector (Amersham Biosciences). The clones were confirmed by DNA sequence. The recombinant plasmids harboring the respective target genes were transformed into *Escherichia coli* BL21 (DE3) host cells individually for protein preparation and the GST fusion protein were purified using Glutathione Sepharose 4B beads (Amersham Biosciences). Protein purity and amounts were checked and normalized by SDS-PAGE and Coomassie staining.

### Blot Overlay Assay

The native core histones H3, H4, H2A, and H2B from calf thymus (Roche) were resolved on a 15% SDS-polyacrylamide gel, which were then transferred to a nitrocellulose membrane (Amersham Bioscience). The membrane was subsequently blocked with 5% skim milk in 50 mM Tris-HCl, pH 7.2, containing 150 mM NaCl and 0.2% Tween-20 for one hour at room temperature, followed by washing with a buffer containing 50 mM Tris-HCl (pH 7.2), 150 mM NaCl and 0.2% Tween-20 at room temperature. The nitrocellulose membranes were then incubated with purified individual GST-BRD in the same buffer for 1 hour at room temperature. After washing the bound GST-BRD was immuno-detected by Western blots with anti-GST antibodies (Amersham Bioscience).

### Dot Blot Assay

Histone binding selectivity of the yeast BRDs was assayed by a dot blot assay. Biotinylated histone acetylated peptides dissolved in 50 mM Tris-HCl pH 7.5 were dotted onto SAM Biotin Capture Membrane (Promega) that is allowed to air dry for 6 minutes. The dot blot overlay assay followed the experimental procedures of the blot overlay assay as mentioned above. The integrated density of each spot on the SAM Biotin Capture membranes was analyzed by Image J software and followed to transfer into 2D matrix using *Matrix2png* [35] software. Empty spots and GST spots were used as controls. For a particular membrane, the average density of 5 empty spots was taken as the background signal, B. The standard deviation of the density of an empty spot ranged between 3–10% from one membrane to the other. For meaningful comparison of intensities across different membranes, the intensity of each spot on a membrane was represented as relative intensity RI = (X_i_-B)/G, where X_i_ is the intensity of the i^th^ point of membrane, and B and G are the baseline intensities of empty spot and GST (5 ng) respectively. However, since there were significant differences between the RI values of GST from one membrane to the other, the RI values of the four subtypes (Figure 2B) were scaled as (Ws) x (RI) where Ws is the scaling factor. We used Ws as 1.00, 0.95, 0.50 & 0.20 respectively for the four subtypes (Figure 2C).

### Comparative Protein Structure Modeling

All known experimental BRD structures (23 X-ray and 3 NMR) were downloaded from the June-2009 PDB release and used as comparative modeling templates for eleven yBRDs. Three-dimensional experimental structures are available for three yBRDs, i.e. yGcn5, yRsc4-1 and yRsc4-2. The templates were superposed using the MALIGN3D command of MODELLER [36] to generate structure-based multiple alignments for modeling the remaining 11 yBRDs. To generate the respective modeling alignments, the sequences of each of the 11 yBRDs were manually adjusted to align to the template structural-alignment, based on consensus obtained from different multiple alignments (MUSCLE, hmmalign, MODELLER sequence-to-structure MALIGN2D) of (26+11) sequences. MODELLER was used to build models. Structural analyses were carried with the modeled structures. Low-resolution electrostatic potential surfaces were generated using PyMol with default parameters.

### Molecular Interaction Field Characterization of Yeast BRD Structures

Molecular Interaction Field (MIF) analysis of the BRD structural models was carried out with program GRID [37]. Due to the complexity of the MIF data, Principal Component Analysis (PCA) was used to identify regions of the binding site that account for most of the MIF variation in the yeast BRD family. PCA is an established statistical procedure to analyze complex datasets and capture their main features. PCA works by linearly transforming the data to a different coordinate system in such a way that, projecting the dataset onto the coordinates (called Principal Components), the variance is maximal on the first principal component and decreases as we move to the other components. The Principal Components can be used to cluster the data in an unsupervised way and to reduce the complexity inherent to the datasets. The complex datasets generated by computing MIFs for all BRDs with different chemical probes were collected into one matrix, which was analyzed using PCA. The first principal component (PC1) was used to cluster the yeast BRDs using a neighbor-joining algorithm. The loadings of PC1 were used to identify the regions in the BRD structures that contribute to the PC1-based clustering.

## Supporting Information

Figure S1 Binding of yeast BRDs to histone peptides. Binding of yeast BRDs to various histone peptides as evaluated by NMR. Superposition of 2D 1H-15N HSQC spectra of individual yeast BRDs in the free form (black signals) and in the presence of a lysine-acetylated (right column) or non-acetylated (left column) histone peptide (red signals) derived from known acetylation sites. The protein concentration was ∼0.25 mM, and the molar ratio of protein∶peptide was ∼1∶5.
(3.49 MB TIF)

Figure S2 Electrostatic potential surfaces of bromodomains. Comparison of electrostatic potential surfaces of (A) experimentally determined structures of BRDs that are known to bind to lysine-acetylated peptides; (B) modeled structures of yBRDs that are shown to interact with histone peptides (G group); and (C) modeled structures of yBRDs that do not show to bind to histone peptides (B group).
(7.95 MB TIF)
