# Supplementary figures and images for: Biochemical Profiling of Histone Binding Selectivity of the Yeast Bromodomain Family
Source: PLoS One. 2010 Jan 26;5(1):e8903. doi: 10.1371/journal.pone.0008903 (PMC2811197; doi:10.1371/journal.pone.0008903)

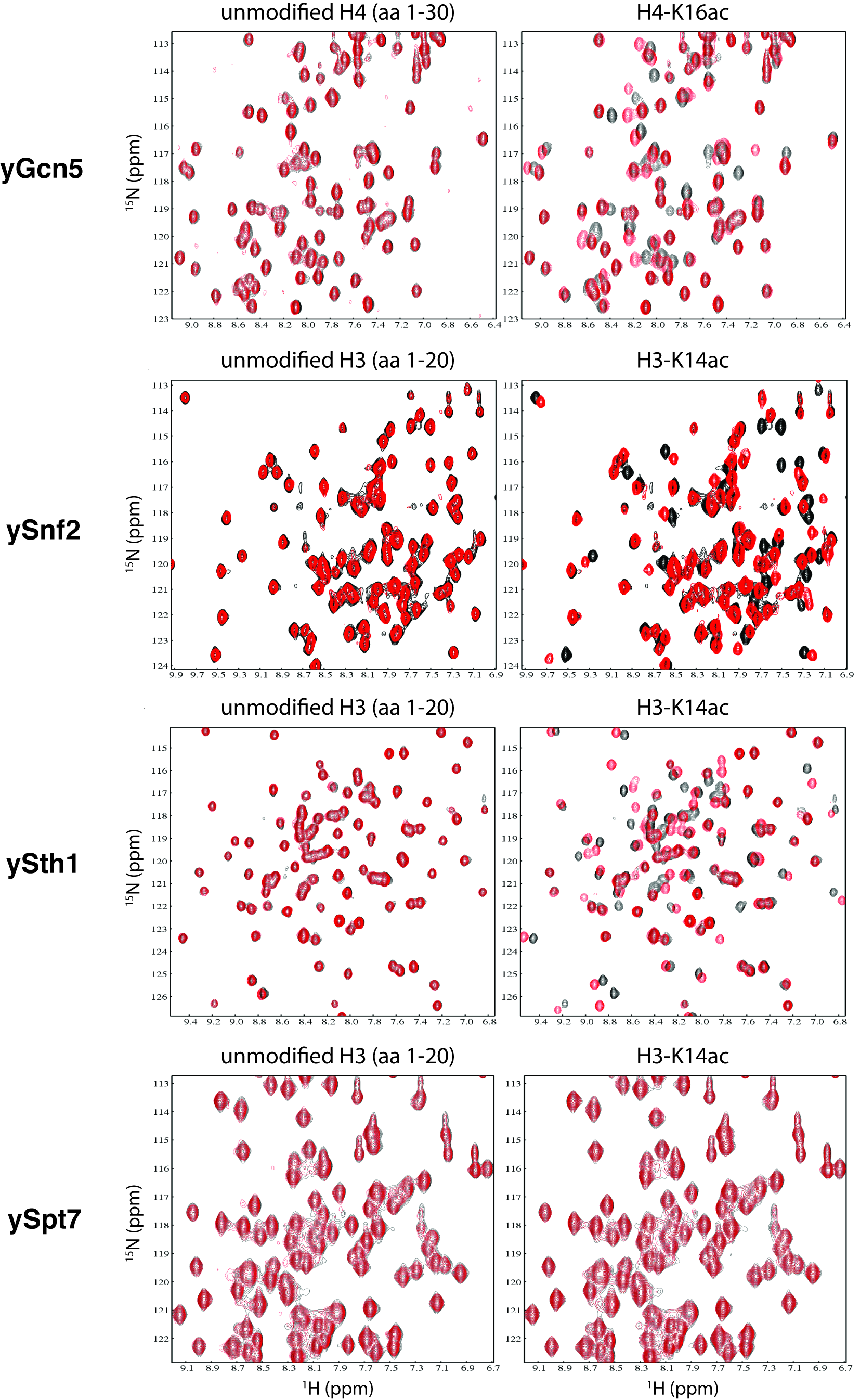

Supplement: Figure S1 — Binding of yeast BRDs to histone peptides. Binding of yeast BRDs to various histone peptides as evaluated by NMR. Superposition of 2D 1H-15N HSQC spectra of individual yeast BRDs in the free form (black signals) and in the presence of a lysine-acetylated (right column) or non-acetylated (left column) histone peptide (red signals) derived from known acetylation sites. The protein concentration was ∼0.25 mM, and the molar ratio of protein∶peptide was ∼1∶5. (3.49 MB TIF) [file pone.0008903.s001.tif]

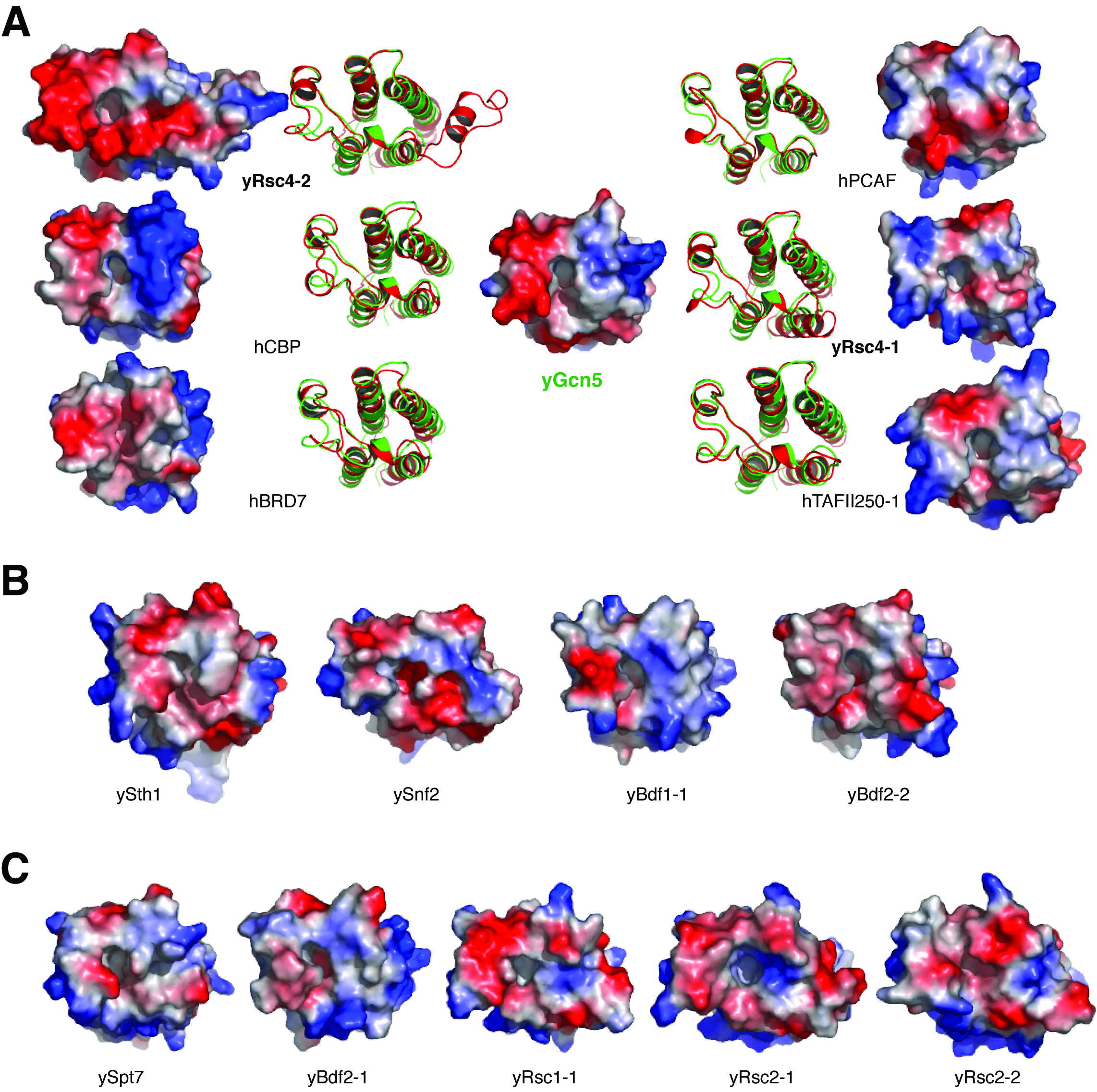

Supplement: Figure S2 — Electrostatic potential surfaces of bromodomains. Comparison of electrostatic potential surfaces of (A) experimentally determined structures of BRDs that are known to bind to lysine-acetylated peptides; (B) modeled structures of yBRDs that are shown to interact with histone peptides (G group); and (C) modeled structures of yBRDs that do not show to bind to histone peptides (B group). (7.95 MB TIF) [file pone.0008903.s002.tif]
